# Contribution of mechanosensitive channels to osmoadaptation and ectoine excretion in *Halomonas elongata*

**DOI:** 10.1007/s00792-020-01168-y

**Published:** 2020-04-07

**Authors:** Jasmina Vandrich, Friedhelm Pfeiffer, Gabriela Alfaro-Espinoza, Hans Jörg Kunte

**Affiliations:** 1 Division Biodeterioration and Reference Organisms, Bundesanstalt für Materialforschung und -prüfung, Berlin, Germany; 2 Computational Biology Group, Max-Planck-Institute of Biochemistry, Martinsried, Germany; 3 Present Address: Research Coordination, German Federal Institute for Risk Assessment, Berlin, Germany; 4 Present Address: Systems and Synthetic Metabolism Group, Max-Planck-Institute of Molecular Plant Physiology, Potsdam, Germany

**Keywords:** *Halomonas elongata*, Ectoine, Mechanosensitive channel, MscS, MscK, Osmoadaptation, Solute excretion, Osmotic shock

## Abstract

For osmoadaptation the halophilic bacterium *Halomonas elongata* synthesizes as its main compatible solute the aspartate derivative ectoine. *H. elongata* does not rely entirely on synthesis but can accumulate ectoine by uptake from the surrounding environment with the help of the osmoregulated transporter TeaABC. Disruption of the TeaABC-mediated ectoine uptake creates a strain that is constantly losing ectoine to the medium. However, the efflux mechanism of ectoine in *H. elongata* is not yet understood. *H. elongata* possesses four genes encoding mechanosensitive channels all of which belong to the small conductance type (MscS). Analysis by qRT-PCR revealed a reduction in transcription of the *mscS* genes with increasing salinity. The response of *H. elongata* to hypo- and hyperosmotic shock never resulted in up-regulation but rather in down-regulation of *mscS* transcription. Deletion of all four *mscS* genes created a mutant that was unable to cope with hypoosmotic shock. However, the knockout mutant grew significantly faster than the wildtype at high salinity of 2 M NaCl, and most importantly, still exported 80% of the ectoine compared to the wildtype. We thus conclude that a yet unknown system, which is independent of mechanosensitive channels, is the major export route for ectoine in *H. elongata*.

**Electronic supplementary material:**

The online version of this article (10.1007/s00792-020-01168-y) contains supplementary material, which is available to authorized users.

## Introduction

To allow for survival in high saline environments, many Bacteria and Archaea accumulate organic compounds like sugars, amino acids, and/or their derivatives to serve as osmolytes (Galinski 1995; Roberts 2005; Widderich et al. 2016). These highly water-soluble molecules do not interfere with the cell’s metabolism and are, therefore, called compatible solutes (Brown 1976). Cells can amass compatible solutes in their cytoplasm in molar concentrations either by de novo synthesis (Galinski 1995; Klähn and Hagemann 2011; Kuhlmann and Bremer 2002; Peters et al. 1990) or by uptake from the medium through osmoregulated transporters (Farwick et al. 1995; Kempf and Bremer 1998; Kunte 2006; Mikkat et al. 1997; Wood 1999; Wood et al. 2001, 2005). A sudden decrease in salt concentration (e.g., rainfall) will lead to a massive water influx into the cell, with a concomitant increase in cell turgor. To avoid cell disruption, the cytoplasmic membrane contains mechanosensitive channels, which act as emergency valves (Martinac 2001). These mechanosensitive channels are stretch-activated pores and open in response to an increase in membrane tension. Their opening allows for a rapid, non-specific release of cytoplasmic, low molecular-weight solutes. Different classes of mechanosensitive channels exist in various bacteria, which are defined according to their pore size and gating behavior (Cox et al. 2018). The most important mechanosensitive channels are the channels of large (MscL) and small (MscS) conductivity and were originally described in *Escherichia coli* (Berrier et al. 1996). The MscS family is more widely spread in Bacteria (Pivetti et al. 2003) and many halophilic bacteria possess no mechanosensitive channels of the MscL-type. This led to the speculation that the loss of MscL is an adaptation to saline environments (Penn and Jensen 2012).

The halophilic gamma-proteobacterium *Halomonas elongata* can tolerate salt concentrations well above 1.7 M NaCl (100 g/L) (Vreeland et al. 1980) and accumulates the compatible solute ectoine for osmoadaptation (Göller et al. 1998; Ono et al. 1999). Interestingly, ectoine functions not only as an osmoregulatory compatible solute but also protects cell components (membranes, proteins, DNA) and even whole cells against different stressors such as freezing and thawing, high temperatures, drying and UV-light (Bünger et al. 2001; Kolp et al. 2006; Lippert and Galinski 1992; Zaccai et al. 2016). It also protects lung epithelia against nanoparticle-induced inflammation (Sydlik et al. 2009), and damage to the small bowel from ischaemia and reperfusion injury (Wei et al. 2009). Recently, it was shown that ectoine is a potent protector of DNA against ionizing radiation (Schröter et al. 2017). Its protective properties make ectoine a valuable compound and ectoine is marketed in health care and skin care products worldwide (Kunte et al. 2014; Lentzen and Schwarz 2006). Thus, ectoine is produced annually on a scale of tons by industry in a biotechnological process with *H. elongata* used as producer strain (Kunte et al. 2014).

*Halomonas elongata* amasses ectoine in the cytoplasm either by synthesis via a pathway utilizing enzymes specified by three genes, *ectABC* (Göller et al. 1998), or by uptake via the ectoine-specific osmoregulated transporter TeaABC (Grammann et al. 2002; Kuhlmann et al. 2008; Schweikhard et al. 2010). TeaABC is not only required for the osmoregulatory accumulation of external ectoine, but also counterbalances a mechanism responsible for excreting endogenous ectoine from the cell (Grammann et al. 2002; Kunte 2006). The mechanism was discovered based on the finding that mutant strains of *H. elongata* with an inoperable TeaABC transporter constantly release ectoine to the surrounding medium (Grammann et al. 2002; Kunte et al. 2002). Still these mutants can keep the cytoplasmic ectoine concentration at the same level as the wild-type strain. Apparently, the mutation of *teaABC* not only causes ectoine excretion to the medium but results in overproducing ectoine. This observation led to the hypothesis that TeaABC-mediated ectoine uptake and the release of internal ectoine is linked to regulating ectoine synthesis (Kunte 2006). However, the mechanism by which ectoine is released from the cell is yet unknown.

Börngen et al*.*, working on *Corynebacterium glutamicum*, presented results that argued for a mechanism of fine-tuning the internal compatible solute concentration of glycine-betaine by cooperation of an active uptake via transporter BetP and a passive, but regulated efflux via mechanosensitive channels of the MscS-type (Börngen et al. 2010). However, the MscS variant of *C. glutamicum* differs structurally from MscS of other bacteria and it is, therefore, debated whether the export via mechanosensitive channels (MS channels) in bacteria is a wide-spread mechanism in fine-tuning the cell’s compatible solute concentration. In contrast to *C. glutamicum*, none of the known MscL- and MscS-type of MS channels in *B. subtilis* participate in export of the endogenous compatible solute proline (Hoffmann et al. 2012). Moreover, in osmotically adapted *E. coli* cells export of the compatible solutes ectoine and hydroxyectoine is also independent of MS channels (Czech et al. 2016).

*Halomonas elongata* possesses four genes encoding MS channels, which all belong to the MscS-family. The objective of this research was to determine the role of MS channels for osmoadaptation in *H. elongata* and in excreting ectoine from the cell. We performed transcriptional analyses of the MS channel genes and generated a mutant, which lacks all MS channel genes. Subsequently, each of the individual genes was subjected to analysis after reinsertion into the genome. These genetic modifications were performed in parallel in wildtype and in the *teaABC* deletion mutant. Our results show that mechanosensitive channels play only a minor role in ectoine excretion.

## Materials and methods

### Bacterial strains and growth conditions

Bacterial strains are listed in Suppl. Table 1. *Escherichia coli* DH5α cells were grown overnight at 37 °C in Luria–Bertani (LB medium). Generally, all strains of *Halomonas elongata* were grown aerobically at 30 °C in MM63 minimal medium (Larsen et al. 1987) with glucose as the carbon source and at various NaCl concentrations. Growth curves were recorded in a volume of 200 µl in 96 well plates with a plate reader (BioTek Synergy HT). For analyzing ectoine and glucose concentration in growth medium, *H. elongata* KB2.13 and KB2.13-MSC were grown in MOPS minimal medium (0.05 M glucose, 0.07 M NH_4_Cl, 0.017 M KH_2_PO_4_, 0.02 M K_2_SO_4_, 0.1 M MOPS, 5 µM Na_2_-EDTA, 3.2 mM MgSO_4_, 0.1 mM FeSO_4_, NaCl as indicated in the experimental setting, pH 7).

### Salt-shock experiments

*Halomonas elongata* was grown in MM63 minimal medium supplemented with 1 M NaCl until OD_600_ reached 0.8. For hypoosmotic and hyperosmotic shock, the cultures were diluted sixfold in fresh media to 0.1 M NaCl or 2 M NaCl, respectively. Cell viability was monitored by constant recording of the OD_600_ and counting of colony forming units in spotting assays.

### Construction of vectors for gene deletion or insertion

For gene deletion, DNA sequences 1 kb upstream and downstream from the gene of interest were amplified with 20 nt overhangs and fused into the shuttle vector pK18*mobsacB* or pK19*mobsacB* (Schäfer et al. 1994) applying Gibson assembly according to the manufacturer’s instruction (New England Biolabs). For gene insertion the gene of interest plus 1 kb upstream and downstream were amplified and fused into the shuttle vector with Gibson assembly. All vectors are listed in Suppl. Table 2 and the corresponding primers used for the amplification of fragments are listed in Suppl. Table 3. The primers M13fwd and M13rev were used to check the inserts by PCR amplification and DNA sequencing.

### Generation of deletion and insertion mutants

Plasmid pK18*mobsacB* or pK19*mobsacB* carrying the corresponding DNA for deletion and insertion, respectively, were transformed into *E. coli* ST18. The plasmids were transferred into *H. elongata* via biparental conjugation (Alfaro-Espinoza and Ullrich 2015; Kunte and Galinski 1995). Fresh overnight cultures of *E. coli* ST18, carrying the corresponding plasmid, and of *H. elongata* were mixed in a ratio of 1:2 and 20 µl drops were spotted on LB agar plates supplemented with l-aminolevulinic acid (Sigma-Aldrich). After 24 h incubation at 30 °C, cells were scratched from the plate, resuspended in LB medium and vortexed for 10 min. The cell suspension was diluted, plated on LB medium plus the respective antibiotic and incubated overnight at 30 °C. Deletion mutants, arising after double cross-over, were then selected for on LB medium containing 22% (w/v) sucrose at 37 °C. The deletion and insertion sites were verified by PCR and DNA-sequencing.

### RNA extraction

RNA extraction was performed with RP-CTAB buffer (3 mM EDTA, 700 mM NaCl, 40 mM DTT) and hot SDS-buffer (3 mM EDTA, 700 mM NaCl, 2% (w/v) SDS) followed by Phenol–Chloroform extraction (Schenk et al. 2008). After extraction, the RNA was treated with TURBO DNA-free Kit (Life technologies).

### qRT-PCR

Gene transcription analysis was performed using the iTaq™ Universal SYBR^®^ Green One-Step Kit on Stratagene Mx3005P Quantitative PCR System. Three biological and three technical replicates were used per treatment. All qRT-PCR primers (Suppl. Table 4) were selected to keep amplification efficiency in an optimum range of 95–105%. Normalized relative expression (NRE) values were calculated according to the PFAFFL method (Pfaffl 2001) in relation to the reference gene *recA*. Gene *recA* was chosen from 8 putative reference genes (16S, *gyrA*, *gyrB*, *lysA*, *recA*, *rho*, *rpoA*, *rpoB*) after testing for stable expression, suitability and efficiency according to published recommendations of Kozera and Rapacz (2013) and based on the proteome data of *H. elongata*, which were collected from cells grown at various salt concentrations (Kindzierski et al. 2017). The one-way ANOVA (ANalysis Of VAriance) with post-hoc Tukey HSD (Honestly Significant Difference) test was used to evaluate significance.

### Glucose quantification

The dinitrosalicyclic acid method (Miller 1959) has been applied to 200 µl volume in microtiter plates. 100 µl dinitrosalicylic acid reagent (1% (w/v) dinitrosalicylic acid, 1.5% (w/v) NaOH, 40% (w/v) potassium sodium tartrate, 2% (w/v) phenol, 0.5% (w/v) Na_2_SO_4_) was added to 100 µl of cell culture supernatant. The mixture was heated at 95 °C for 5 min and the OD_575_ was recorded with a plate reader (BioTek Synergy HT).

### HPLC analysis of ectoine

For HPLC analysis, *H. elongata* was grown at 33 °C, shaking with 220 rpm in MOPS minimal medium with NaCl concentrations as indicated in the experimental setting. The samples were centrifuged for 5 min at 4 °C and 4000×*g*. The supernatant was filtered to remove cell debris and then analyzed without further dilution or treatment. The ectoine content of the samples was measured by ultra-high-performance liquid chromatography (U-HPLC) on an Agilent Technologies 1290 Infinity HPLC-system using a reverse-phase column (GromSil 100 Amino-1 PR, 3 µm) and applying an acetonitrile/H_2_O gradient as mobile phase. The absorption of ectoine was recorded at 207 nm using an UV-detector (Kuhlmann and Bremer 2002).

### Bioinformatic analyses

There are four annotated mechanosensitive channels in the *H. elongata* genome (*mscS*: HELO_3171, HELO_3378, HELO_4248; *mscK*: HELO_2045) (Pfeiffer et al. 2017; Schwibbert et al. 2011). We made two attempts to search for additional, yet unannotated mechanosensitive channels. (a) We downloaded the entry for each mechanosensitive channel of *Escherichia coli* (mscS-related: UniProt P0C0S1 mscS, P77338 mscK, P39285 mscM, POAEB5 ynaI, P75783 ybiO, P0AAT4 mscM; mscL-related: P0A742 mscL) (Berrier et al. 1996) and of *Corynebacterium glutamicum* (Cgl0879 mscL UniProt:Q8NS07; Cgl1270 yggB UniProt:P42531; KIQ_000100 UniProt:A0A072ZAU2) (Börngen et al. 2010; Nottebrock et al. 2003) for subsequent BLASTp analyses. All BLASTp results either retrieved the already annotated mechanosensitive channels or did not identify homologs. (b) We searched in InterPro (https://www.ebi.ac.uk/interpro) for “mechanosensitive” and retrieved 16 InterPro domains related to mechanosensitive channels (plus the domain from one toxin that inactivates mechanosensitive channels). The domain codes were then used to search the *H. elongata* proteome in UniProt. Of these, 8 domains (IPR006685, IPR023408, IPR011066, IPR011014, IRP006686, IPR025692, IPR024393, IPR008910) retrieved the 4 annotated mechanosensitive channels (or subsets thereof). One domain (IPR010920, “LSM domain superfamily”) retrieved the same set plus, in addition, Hfq (HELO_3364). This is the expected behavior according to the InterPro domain annotation. The other 7 domains (IPR036019, IPR001185, IPR019823, IPR037673, IPR016688, IPR030192, IPR031334) did not retrieve entries from *H. elongata*. From this analysis, we concluded that the exhaustive set of mechanosensitive channels of *H. elongata* is represented by the four genes which are annotated accordingly.

## Results

### Identification of MS channels in *H. elongata*

In the genome of *H. elongata*, four MS channels are annotated. Three are annotated as *mscS* and one as *mscK* (Table 1, Suppl. Figures 1, 2, 3). MscK was formerly annotated as potassium efflux system KefA (Cox et al. 2015). Additional MS channels could not be identified by BLASTp analysis with MS channel proteins from *Corynebacterium glutamicum* (Börngen et al. 2010; Nottebrock et al. 2003) and from *E. coli* (Berrier et al. 1996). In addition, no additional *H. elongata* proteins contain any of the InterPro domains, which are associated with MS channels. Thus, the analysis of the four MS channels as listed in Table 1 was considered to be exhaustive.

**Table 1:** Mechanosensitive channels in *H. elongata*

Gene	Code	Length	MscS core region	Homolog	Extra InterPro domains
mscK	Helo_2045	1173	873–1127	MCSK_ECOLI (37%)	IPR024393; IPR025692
mscS1	Helo_3378	281	16–268	MSCS_ECOLI (43%)	–
mscS2	Helo_4248	780	465–718	YBIO_ECOLI (36%)	none
mscS3	Helo_3171	770	467–723	Helo_4248 (28%)	none

In the column “Homolog” the best full-length homolog from the analyzed set of proteins is given, with protein sequence identity indicated in parenthesis. Helo_3171 is more closely related to Helo_4248 than to any of the MscS-related proteins from *E. coli*. All four *H. elongata* proteins have the MscS core region as indicated. For an alignment of this region see Suppl. Figure 1. Most of this region is covered by the InterPro MscS core domain (IPR006685, “MscS_channel”). This domain is split into three consecutive subdomains (IPR011014 “MscS_channel_TM-2”; IPR010920 “LSM_dom_sf”; IPR011066 “MscC_channel_C”). The region covered by LSM may have two additional domain assignments (IPR006686 “MscS_channel_CS”; IPR023408 “MscS_dom_sf”). MscK has additional domains in the N-terminal region (“Extra InterPro domains”: IPR024393 “MscS_porin”; IPR025692 “MscS_IM_dom1”) (alignment in Suppl. Figure 3). The N-terminal regions of Helo_4248 and Helo_3171 (ca 460 aa) are homologous but have no InterPro domain assigned (“none”) (alignment in Suppl. Figure 2)

### Transcription levels of MS channel genes at optimum, low, and high salt

**Fig. 1:**
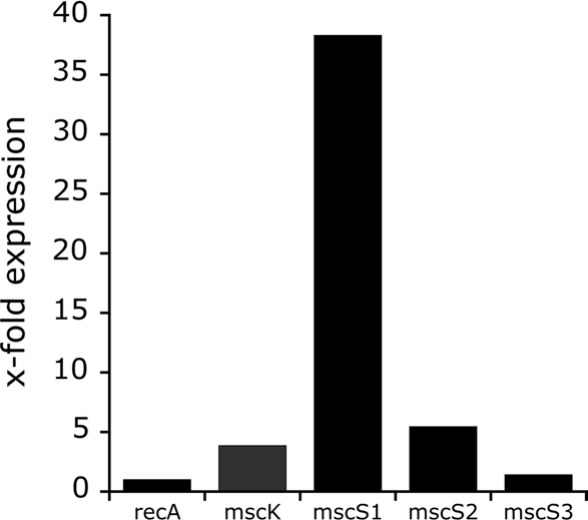
Transcripts of MS channel genes *mscK*, *mscS1*, *mscS2*, and *mscS3* from *H. elongata* cells grown in MM63 minimal medium at 1 M NaCl. Transcript levels are depicted as x-fold expressions in relation to the reference gene *recA.* Data presented are from three independent experiments (three biological replicates). Each experiment was measured three times (three technical replicates)

We quantified transcription levels of all MS channel genes by qRT-PCR with expression of *recA* as a reference. First, transcription was accessed for cells grown at optimum conditions of 1 M NaCl in minimal medium (Fig. 1). The *mscS1* gene was strongly expressed (38-fold in relation to *recA*), while only moderate to small amounts of transcript were found for *mscS2* (fivefold), *mscK* (fourfold) and *mscS3* (onefold).

**Fig. 2:**
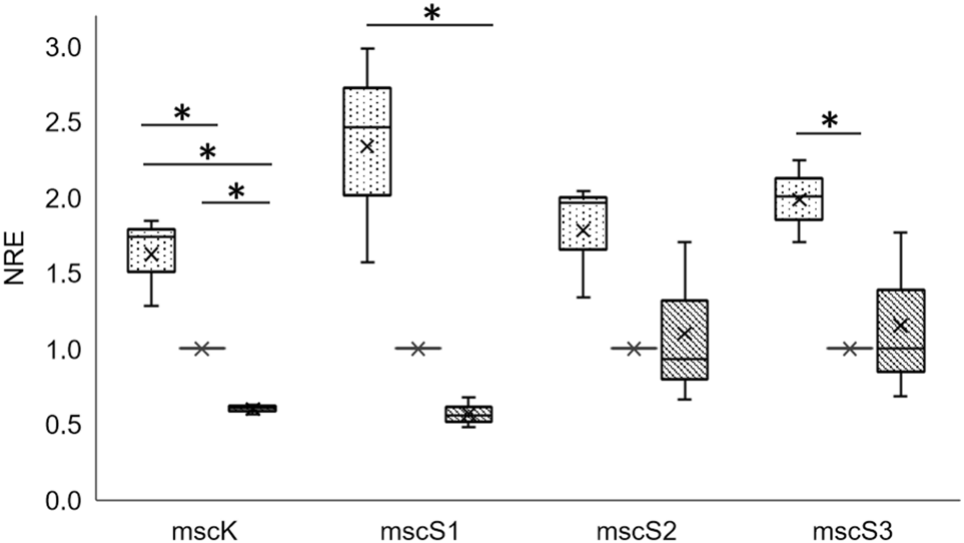
Relative transcription levels of MS channel genes depending on the salinity of the medium. *H. elongata* wildtype was grown in MM63 with 0.1 M NaCl (light grey) and MM63 with 2 M NaCl (dark grey). Results are depicted in relation to the optimum salt concentration of 1 M NaCl (set to 1). Data presented are from three independent experiments (three biological replicates). Each experiment was measured three times (three technical replicates). Asterisk mark significance (*p* value < 0.05) between samples

Next, we determined transcription levels from cells grown at low, medium and high salt conditions. For low salt stress, cells were grown at 0.1 M NaCl, which is below the lower range of preferable salt requirements of 1 M NaCl for *H. elongata*. For high salt stress, cells were grown at 2 M NaCl. At low salt stress conditions, transcription of *mscS3* and *mscK* was significantly upregulated compared to optimal salinity of 1 M NaCl (Fig. 2). In addition, for *mscS1* and *mscS2*, higher transcription values were measured at low salt, but significance was not reached. Comparing transcription at low salt to high salt-stress, both, *mscK* and *mscS1*, were significantly upregulated at low salt (Fig. 2). Taken together, and surprisingly, transcription of MS channel genes tends to decrease with increasing salt concentrations.

Next, we were interested in transcriptional changes of MS channel genes upon osmotic shock. When salt concentration rises, potassium is pumped into the cells of *H. elongata* via TrkH and TrkI transporters and compatible solutes are gathered (Kraegeloh et al. 2005; Kraegeloh and Kunte 2002). When cells are subsequently exposed to a rapid downshift in salinity, the influx of water may cause an increase of turgor until bursting, unless the cell is able to discharge ions and compatible solute quickly. MS channels are responsible for this unspecific release of small, soluble molecules from the cytoplasm. Transcription of MS channel genes was measured for a downshock (hypoosmotic shock) from 1 to 0.1 M NaCl but also for an upshock (hyperosmotic shock) in salinity from 1 to 2 M NaCl. The transcription levels were recorded 30 min and 15 min before the shock and 15 min and 30 min after the shock. In case of an osmotic downshock from 1 to 0.1 M NaCl, the amount of transcripts from all four channels remained unchanged (Table 2, Suppl. Figure 4). In contrast, the three MS channels genes *mscK*, *mscS1* and *mscS3* responded with downregulation to a hyperosmotic shock (Table 2, Suppl. Figure 4), which is in agreement with downregulation observed in adapted cell for *mscS1* and *mscK* (Fig. 2). Gene *mscS2* was not significantly downregulated after upshock from 1 to 2 M NaCl.

**Table 2:** Transcriptional regulation of MS channel genes in response to osmotic shock

	Hypoosmotic shock from 1 to 0.1 M NaCl	Hyperosmotic shock from 1 to 2 M NaCl
MscK	–	**↓**
MscS1	–	**↓**
MscS2	–	–
MscS3	–	**↓**

(–) no regulation, (***↓***) significant downregulation

For further details see Suppl. Figure 1 in Supplementary Material

### Deletion of all MS channel genes in *H. elongata*

All four genes coding for MS channels were deleted from the *H. elongata* DSM 2581^T^ wildtype genome (see “Material and Methods”). Gene deletions were validated by PCR analysis and DNA sequencing. The resulting mutant strain lacking all MS channel genes was designated *H. elongata* MSC-1324 (∆*mscK,* ∆*mscS1*, ∆*mscS*2, ∆*mscS3*).

### Growth of *H. elongata* wildtype and mutant MSC-1324 at different salt concentrations

It was assessed whether the growth *of H. elongata* MSC-1324 varies from the parental wildtype strain, when grown at different salt concentrations. The deletion of all MS channel genes might influence the growth in extreme salt concentrations due to alterations of the adjustment mechanism for intracellular substances and ectoine concentration.

**Fig. 3:**
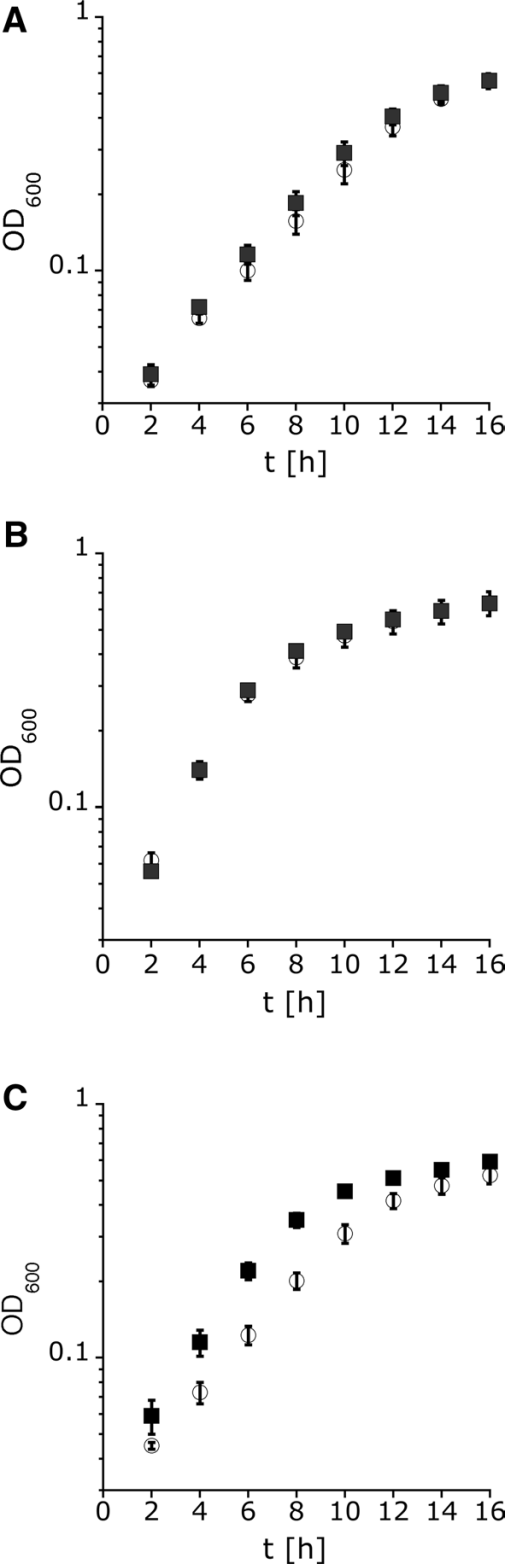
Growth of *H. elongata* MSC-1324 depending on the salinity of the growth medium. Growth of *H. elongata* wildtype (white) and mutant MSC-1324 (black) was assessed at **a** 0.1 M NaCl, **b** 1 M NaCl, and **c** 2 M NaCl in MM63 minimal medium until onset of stationary phase. Data shown are the mean from three biological and 9 technical replicates

The growth rate of the MSC-1324 mutant was very similar to that of the wildtype at low and medium salinity (Table 3; Fig. 3a, b) and at all salinities the wildtype and the MSC-1324 mutant reached the same final optical density. At high salinity of 2 M NaCl, however, MSC-1324 grew significantly faster than the wildtype (Fig. 3c). The growth advantage of mutant MSC-1324 is consistent with our finding that MS channels genes are downregulated in wildtype cells at high salt. Apparently, MS channels pose a burden for salt-stressed cells of *H. elongata* and it is, therefore, meaningful to downregulate them at high salt*.*

**Table 3:** Growth parameters of *H. elongata* DSM 2581^T^ (wildtype) and MS channel mutant MSC-1324

[NaCl]	Growth rate [*h*^−1^]		Generation time [*h*]		Max OD_600_		*t* (max OD_600_) [*h*]	
	Wt	MSC-1324	Wt	MSC-1324	Wt	MSC-1324	Wt	MSC-1324
0.1 M	0.22	0.26	3.09	2.71	1.04	1.04	32	38
1.0 M	0.38	0.41	1.84	1.69	1.40	1.39	60	60
2.0 M	0.24	0.30	2.88	2.33	1.28	1.29	48	48

Growth rate, generation time and maximal optical density at 600 nm (OD_600_) were compared. Both strains were grown in MM63 medium at low (0.1 M NaCl), medium (1 M NaCl), and high salt (2 M NaCl). Data presented are from three independent experiments (three biological replicates). Each experiment was measured 9 times (9 technical replicates)

### Significance of MS channels for adaptation of *H. elongata* to hypoosmotic shock

MS channels are known to act as emergency valves, when cells must cope with sudden dilution of salt water by rainfall or flooding (hypoosmotic shock) (Berrier et al. 1992; Levina et al. 1999; Oren 2002). We investigated the behavior of *H. elongata* MSC-1324 compared to the wildtype following an osmotic downshock and monitored the survival by total cell count (OD_600_) and live cell count. Finally, the contribution of each MS channel for the survival of *H. elongata* was assessed. For this, we restored each single MS channel gene in the quadruple MS channel deletion mutant *H. elongata* MSC-1324. The corresponding MS channel gene was transferred into the mutant strain via the conjugative plasmid pK18*mobsacB* (Schäfer et al. 1994) and placed at its original genomic position by homologous recombination. Correct insertion of the genes was verified by PCR and DNA sequencing.

**Fig. 4:**
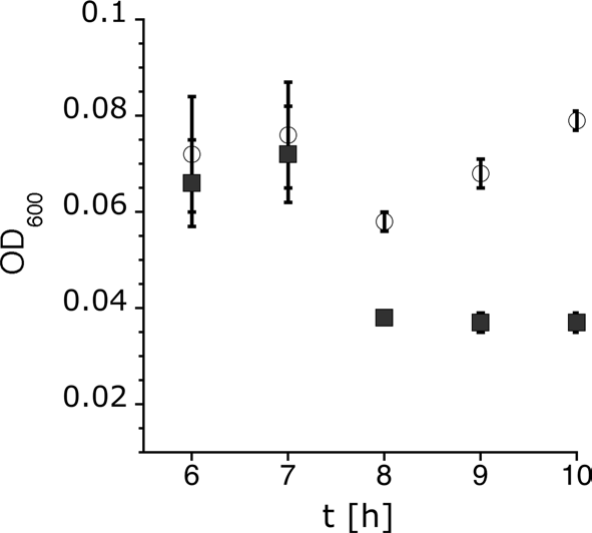
Survival of *H. elongata* wildtype and MS channel mutant MSC-1324 (∆*mscK,* ∆*mscS1*, ∆*mscS*2, ∆*mscS3*) after hypoosmotic shock. The optical density was monitored to trace the survival of wildtype *H. elongata* DSM 2581^T^ white) and mutant *H. elongata* MSC-1324 (black) after an hypoosmotic shock from 1 to 0.1 M NaCl. For osmotic shock, cultures were diluted in fresh media (1:6). The OD values before shock were normalized accordingly. The osmotic shock was applied after seven and a half hours (7.5 h). Data presented are from three independent experiments (three biological replicates). Each experiment was measured three times (three technical replicates)

**Fig. 5:**
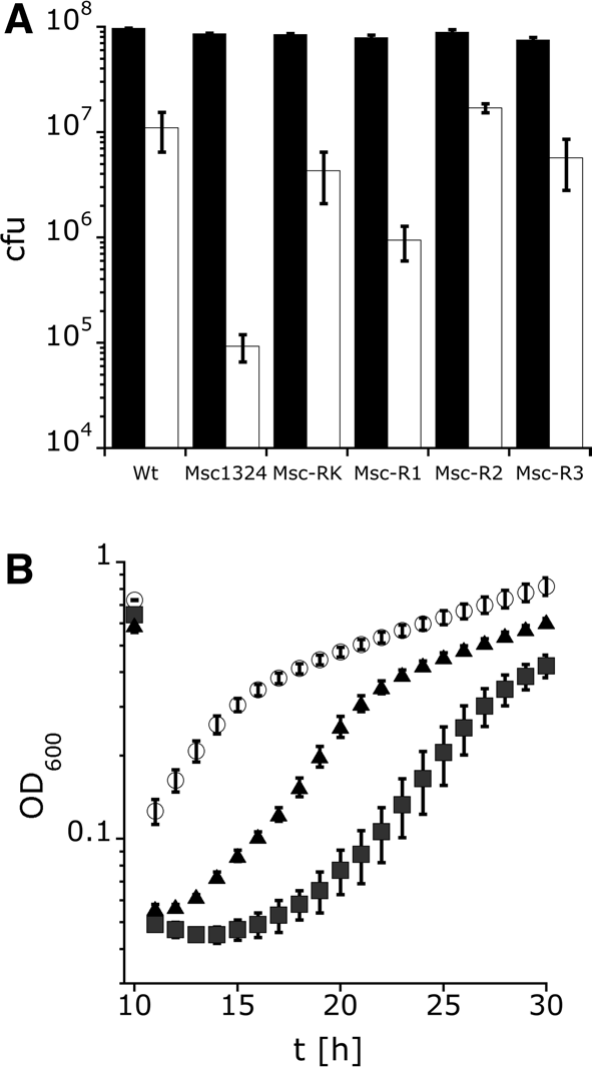
Survival after osmotic shock of *H. elongata* wildtype (wt) compared to *H. elongata* strains carrying only one MS channel gene. **a** Live cells have been counted before (black) and after osmotic shock (white). **b** Recovery of growth after hypoosmotic has been monitored for the *H. elongata* wildtype (white), strain MSC-1324 (black squares) and strain MSC-R1 (black triangles). All other complemented mutants behaved liked the wildtype (data not shown). *H. elongata* MSC-1324 (*∆mscK, ∆mscS1*, *∆mscS2, ∆mscS3*), *H. elongata* MSC-RK (*mscK*^+^*, ∆*mscS1, *∆mscS2, ∆mscS3*), *H. elongata* MSC-R1 (*mscS1*^+^, *∆mscK*, *∆mscS2, ∆mscS3*), *H. elongata* MSC-R2 (*mscS2*^+^*, ∆mscK, ∆*mscS1, *∆mscS3*), *H. elongata* MSC-R3 (*mscS3*^+^*, ∆mscK, ∆*mscS1, *∆mscS2*). Data presented are from three independent experiments (three biological replicates). Each experiment was measured three times (three technical replicates, 5A) and 9 times (5B), respectively

All mutant strains and the wildtype were exposed to hypoosmotic shock. Following hypoosmotic shock, the optical density of the wildtype culture dropped by approximately 25%, while the optical density of the MS channel mutant MSC-1324 dropped by approximately 50% (Fig. 4). Cell counting revealed that 10% of the wildtype cells survived a downshock from 1 to 0.1 M NaCl, but the major part of the mutant cells, which are unable to release the excess of solutes, burst during osmotic downshock (Fig. 5a). Only 0.1% of the mutant cells survived such a downshock. The wildtype recovered quickly and proceeded to grow. Growth of the mutant halted for the entire observation time after the shock (Fig. 4).

We next analyzed the survival after hypoosmotic shock and growth behavior of the mutants expressing only one of the four MS channel genes to assess the biological function and relevance of each channel. The three MS channels, MscK, MscS2, and MscS3, individually were able to restore the wildtype level of survival, with 10% of the cells surviving hypoosmotic shock (Fig. 5a). Likewise, the mutant cells resumed growth after shock similar to the wildtype (data not shown). However, inserting gene *mscS1* (Helo_3378) resulted in only partial complementation and the mutant strain was still severely impaired in stress adaptation. Only 1% of the cells survived the shock. The surviving cells also recovered significantly slower compared to the wild type (Fig. 5b) and compared to the complemented strains carrying any of the other MS channel genes. Taken together, reinserting the MS channel genes restored the wild type phenotype partially (*mscS1*) or completely (*mscK, msc2, msc3*), which proved that all four genes are functional in *H. elongata.*

### Deletion of all MS channel genes in ectoine excretion mutant KB2.13

The ectoine excretion strain KB2.13 (∆*doeA,* ∆*teaABC*) (Schwibbert et al. 2011) is leaking ectoine continuously to the medium. Strain KB2.13 is deficient in osmoregulatory uptake of ectoine (∆*teaABC*) and unable to degrade ectoine (∆*doeA*), which contributes to an increased ectoine excretion. All four MS channel genes were deleted in strain KB2.13, identically to the procedure in the wildtype strain (see above). As before, the mutation sites were checked by PCR and DNA sequencing. The quadruple MS channel mutant derived from KB2.13 was named *H. elongat*a KB2.13-MSC (∆*doeA,* ∆*teaABC,* ∆*mscK,* ∆*mscS1*, ∆*mscS*2, ∆*mscS3*).

### Impact of MS channel deletion on ectoine excretion

**Fig. 6:**
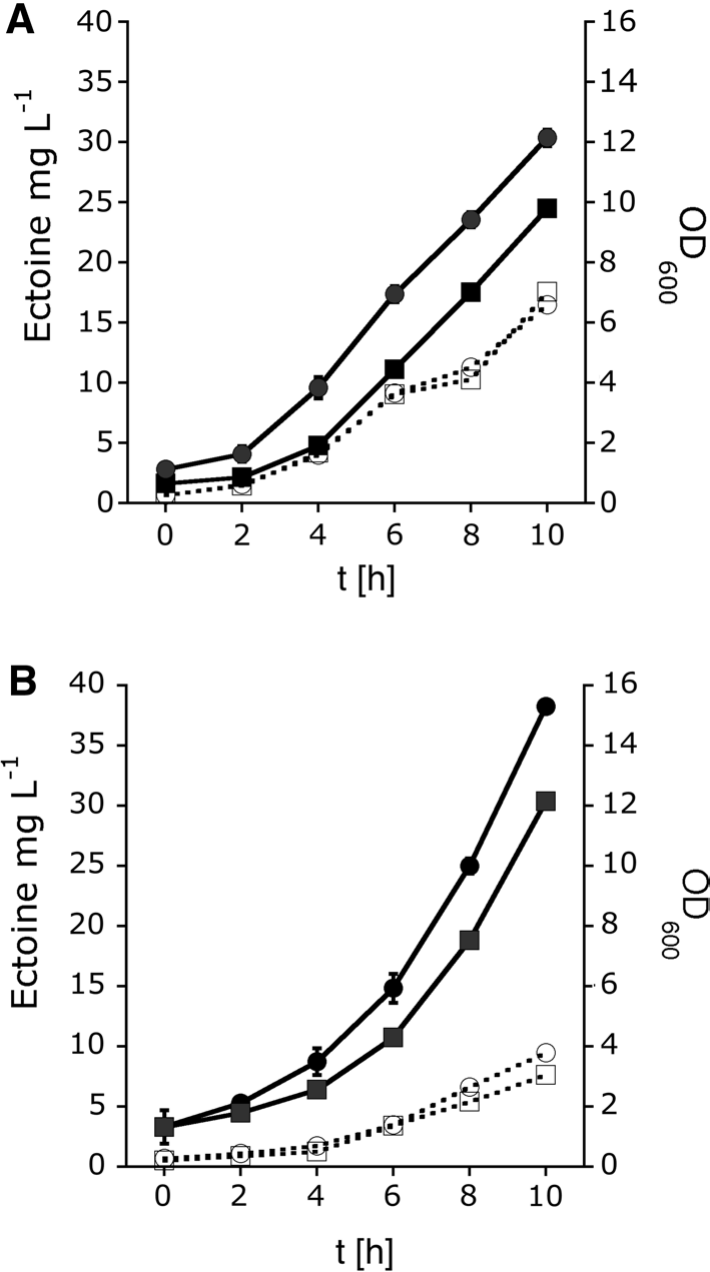
Ectoine excretion from MS channel mutant KB2.13-MSC compared to parental strain KB2.13 at salinities of 0.7 M NaCl (**a**) and 2 M NaCl (**b**). Ectoine concentration in growth medium was measured from ectoine excretion strain KB2.13 (black circle) and KB2.13-MSC (black square) and growth for KB2.13 (white circle) and KB2.13-MSC (white square) was monitored (OD_600_) for 10 h. Mutant strain KB2.13-MSC, which lacks all MS channel genes, still exported approximately 80% of the ectoine compared to KB2.13. Data presented are from three independent experiments (three biological replicates). Each experiment was measured three times (three technical replicates)

Ectoine excretion and glucose consumption were measured for *H. elongata* KB2.13 and the deletion mutant KB2.13-MSC which is devoid of all MS channels. When grown in MOPS medium at 0.7 M and 2 M NaCl, respectively, the strains KB2.13 and KB2.13-MSC consumed identical amounts of glucose and displayed the same growth behavior. The MS channel mutant KB2.13-MSC exported less ectoine to the growth medium during the 10-h experiment. However, only a relatively small reduction of the final ectoine concentration was observed, even though all MS channels had been deleted. The excretion of ectoine was only 19% (at 2 M NaCl) and 21% (at 0.7 M NaCl) lower than for the parental strain (Fig. 6). Thus, even though MS channels of *H. elongata* are an export route for ectoine, they are only of minor importance for this process. Approximately 80% of the ectoine was released from the cell via a major, yet enigmatic pathway.

## Discussion

MS channels play an essential role in the regulation of turgor in bacteria (Krämer 2010). *H. elongata* can maintain turgor pressure across a wide range of osmolarities. The maintenance of turgor pressure requires bacteria to adjust their internal solute pools in response to changes in osmolarity (Booth and Louis 1999; Morbach and Kramer 2002; Wood et al. 2001). Increased expression of transport systems and enzymes ensures that high concentrations of compatible solutes can be established and maintained. We set out to determine the role of mechanosensitive channels for osmoadaptation and for excretion of the compatible solute ectoine in *H. elongata*. For this purpose, we analyzed the transcription of MS channel genes, ectoine excretion and growth behavior in the wildtype strain and a deletion mutant lacking all MS channels under steady state conditions and during osmotic shock.

*H. elongata* possesses in total four genes encoding MS channels, which all belong to the MscS-family. The main role assigned to MS channels in cell physiology is to act as emergency valves upon hypoosmotic shock releasing excess of organics solutes and ions to the medium (Levina et al. 1999). Stokes and co-workers stated that in cells exposed to hypoosmotic shock, MS channels must be activated on a millisecond time scale to prevent damage to cell integrity (Stokes et al. 2003). De novo gene expression cannot modulate the levels of MS channel proteins on this time scale, suggesting that MS channel expression might be induced when cells are exposed to high osmolarity to prepare for the eventuality of hypoosmotic stress (Stokes et al. 2003). However, our results revealed that MS channel expression in *H. elongata* is regulated differently. We assessed the transcript levels in osmotically adapted cells at low (0.1 M), medium (1 M) and high salt (2 M) and observed that the MS channel genes *mscS3* and *mscK* were downregulated, when the salt concentration increased from 0.1 to 1 M NaCl and *mscK* was even further downregulated when salinity reached 2 M NaCl. Gene *mscS1* was downregulated when salinity increased to 2 M NaCl, while changes in transcription of *mscS2* did not reach significance. It must be stressed that the transcription of *mscS2*, *mscS3* and *mscK* is low compared to *mscS1* at 1 M NaCl (Fig. 1). Quantitative analysis of the membrane proteome of *H. elongata* performed by Raschke uncovered a decreased protein content of MscK, MscS1 and MscS3 at high salinity (Raschke 2020), which supports our findings. The proteome and transcription data collected for *H. elongata* do not support the idea that MS channel expression rises with salinity to better prepare the cells for the eventuality of hypoosmotic shock caused, for instance, by rainfall. Our analysis of transcription levels upon osmotic shock supports the observations made with salt-adapted cells. The MS channel genes were downregulated or not regulated at all after hyperosmotic shock and upregulation was never observed.

The finding that the *mscS* genes are rather downregulated is also in agreement with the growth behavior of the MS channel mutant of *H. elongata*. Mutant MSC-1324, which is lacking all MS channels, grew faster at higher salinity compared to the wildtype. Here, the lack of all MS channels seems beneficial and might save energy, which is otherwise required to balance ion and osmolyte flow over the membrane via MS channels.

The importance of MS channels for osmoadaptation in *H. elongata* was demonstrated by hypoosmotic shock from 1 to 0.1 M NaCl, where 100 times less MSC-1324 mutant cells survived compared to the wildtype. In comparison to other bacteria, however, wildtype cells of *H. elongata* have a low survival rate of 10%. The downshock we applied was corresponding to the withdrawal of 900 mM NaCl. *B. subtilis* proved rather resilient and roughly 100% cells survived a shock equivalent to the withdrawal of 760 mM NaCl (Hoffmann et al. 2008). And approximately 90% of *E. coli* cells survived shocks equivalent to the withdrawal of 300–500 mM NaCl (Levina et al. 1999). *C. glutamicum* seems to be more susceptible to hypoosmotic downshock, but still 25% of the cells stayed alive after withdrawal of 750 mM NaCl (Nottebrock et al. 2003). Whether the different cell wall design (e.g., *B. subtilis*) or the lesser stress being applied resulted in the better survival of the non-halophiles is a matter of speculation. It must be noted that after downshock, *E. coli, C. glutamicum* and *B. subtilis* ended up in favorable osmotic conditions, while the withdrawal of NaCl imposed an additional stress of low salt (0.1 M NaCl) on cells of *H. elongata*.

When bacterial cells are adapting to a hyperosmotic environment, compatible solutes like ectoine are accumulated internally to a concentration corresponding to the external osmolality (Bursy et al. 2008; Kuhlmann and Bremer 2002). How the cell matches the internal solute concentration to the external salt concentration is still not quite understood. Important to the understanding of this regulatory problem is the fact that the bacterial cells are releasing compatible solutes to the outside. This observation was made first with *E. coli* cells that were loaded with radioactively labelled glycine-betaine and ectoine, respectively. The radioactive solutes were released from the cell and unlabeled glycine-betaine and ectoine, respectively, were recycled from the medium (Jebbar et al. 1992; Lamark et al. 1992). Subsequently, similar observations were made for other bacteria such as *Synechocystis* sp. (Hagemann et al. 1997). That the release of compatible solutes might be linked to compatible solute synthesis came from the observation made with a *H. elongata* transport mutant. Inactivating TeaABC, the only ectoine uptake system in *H. elongat*a, created an ectoine excretion and ectoine overproducing mutant (Grammann et al. 2002). We concluded that the loss of ectoine and its subsequent uptake by ectoine-specific transporter TeaABC serves as a signal for the regulation of ectoine synthesis. Increasing the ectoine concentration by synthesis will lead to water influx and an increase in turgor pressure, which could trigger stretch-sensitive export channels or carriers for solutes. Transport of exported ectoine back into the cell via TeaABC will downregulate ectoine synthesis. The proposed regulation mechanism would allow for an oscillation of the cytoplasmic ectoine level closely above and below the threshold needed to open the solute-specific export channels and carriers, respectively.

There are in principle four different ways by which compatible solutes can exit the bacterial cell, namely, by (i) passive diffusion across the membrane, (ii) reversal of compatible solute uptake systems, (iii) efflux via (more or less unspecific) channels including MS channels (channel model), and (iv) efflux via carrier systems with either broad or narrow substrate specificity (carrier model). Passive diffusion across the membrane is no plausible mechanism in our view for the release of bacterial compatible solutes. There is also (to our knowledge) no support by proven examples for the release of compatible solutes by reversal of uptake transporters and this can be ruled out for *H. elongata* as the transporter in question (TeaABC) was absent in the investigated strain.

The Krämer group collected evidence that *C. glutamicum* applies a *pump and leak* mechanism in fine tuning the internal glycine betaine concentration by cooperation of an active glycine betaine uptake via osmoregulated transporter BetP and a passive, but regulated efflux via MS channels (Börngen et al. 2010). For releasing the compatible solute glycine-betaine, *C. glutamicum* employs MscCG, a mechanosensitive channel of the MscS-type. MscCG is the major export route for glutamate in *C. glutamicum* and in addition to its osmotic function, it serves as a metabolic valve (Nakayama et al. 2018b). Compared to canonical MscS, it has a long C-terminal extension that has an additional C-terminal transmembrane domain. This extension, including its additional transmembrane domain, is responsible for the unusual gating mechanism of MscCG (Nakayama et al. 2016) with a lower activation threshold and slow closing. In contrast, canonical MscS channels gate in response to hypoosmotic shock and seemingly follow the *Jack-in-the-box* mechanism (Malcolm et al. 2015), which does not fit for MscCG as a consequence of structural adaptations (Nakayama et al. 2019, 2018a).

The export of compatible solutes via channels as described for *C. glutamicum* is not a universal mechanism. Evidence for the existence of compatible solute efflux systems other than channels has been provided for several bacteria. For *Salmonella enterica* (serovar Typhimurium) and *Lactobacillus plantarum* it was shown that compatible solutes are released from the cell by a carrier-mediated process rather than by channels (Glaasker et al. 1996; Koo et al. 1991). *S. enterica* cells loaded with the compatible solute glycine betaine lost glycine betaine upon chemical modification of an unknown efflux system (Koo et al. 1991). Glaasker and coworkers provided evidence for an independent glycine-betaine efflux system in *L. plantarum*, which can be distinguished from MS channels by its kinetic parameters (Glaasker et al. 1996). Upon osmotic downshock, glycine-betaine is rapidly released via a MS channel-like mechanism (osmotic valve) and more slowly by the second system (metabolic valve). The second system is responsible for glycine-betaine efflux in osmotically adapted cells and allows a downhill efflux via a carrier-like mechanism (Glaasker et al. 1996). A channel-independent export of compatible solutes was also described for *B. subtilis* and *E. coli*. In *B. subtilis* none of the known MS channels of the MscL- and MscS-type participated in export of the endogenous compatible solute proline (Hoffmann et al. 2012), and in osmotically adapted *E. coli* cells hydroxyectoine export is independent of MS channels as well (Czech et al. 2016). We are not aware that the export systems have been identified in any of these organisms. However, Bay and Turner (2012) brought forward some indirect evidence (loss-of-growth phenotype under hyperosmotic conditions) that small multidrug resistance (SMR) transporter protein EmrE might be responsible for the export of glycine betaine and choline in *E. coli*.

The data provided by us in the present study draw a similar picture and revealed a MS channel independent export of ectoine from *H. elongata* cells. Specific efflux systems for various types of compounds, in particular amino acids, are already known for different microorganisms (Eggeling and Sahm 2003; Hori et al. 2011; Trötschel et al. 2005). Hence, *H. elongata* might possess a carrier-mediated efflux system, which is either a general compatible solutes exporter or a more specific system for the export of ectoine and eventually hydroxyectoine.

Still the question remains how an efflux system for compatible solutes is linked to osmolarity and can sense the internal solute concentration. For *Dickeya dadantii* (*Erwinia chrysanthemi*) a putative sensor protein was described that enables the cell to specifically measure the internal glycine betaine concentration and the extracellular salt concentration (Touzé et al. 2001). Inactivation of the sensor, which shares some similarities with MscK, resulted in loss of glycine-betaine from the cell but not of other compatible solutes. The ectoine excretion assay, which we developed earlier (Grammann et al. 2002) and was also applied in this study (data not shown), will be a helpful tool in identifying genes encoding such sensors and compatible solute export carries, respectively.

## Electronic supplementary material

Below is the link to the electronic supplementary material.

Supplementary file1 (DOCX 171 kb)
