# Supplementary material for: Contribution of mechanosensitive channels to osmoadaptation and ectoine excretion in Halomonas elongata
Source: Extremophiles. 2020 Apr 7;24(3):421–32. doi: 10.1007/s00792-020-01168-y (PMC7174268; doi:10.1007/s00792-020-01168-y)
Supplement: Supplementary file 1 — Supplementary file1 (DOCX 171 kb) [file 792_2020_1168_MOESM1_ESM.docx]

# **SUPPLEMENTARY MATERIAL**

# Suppl. Table 1

# Bacterial strains

| Species | Strain | Genotype | Reference |
| --- | --- | --- | --- |
| *E. coli* | DH5α | supE44D*lac*U169 (F80 *lacZ*DM15) *hsdR*17 *recA*1 | (1) |
|  | St18 | Δ*pir ΔhemA pro thi hsdR*+ Tpr Smr chromosome::RP4-2 Tc::Mu-Kan::Tn7 | (2) |
| *H. elongata* | DSM 2581^T^ | Wildtype | (3) |
|  | KB2.13 | Δ*teaABC,* Δ*doeA* | (4) |
|  | KB2.13 MSC | Δ*teaABC,* Δ*doeA,* ∆Helo_2045 (*mscK*), ∆Helo_3378 (*mscS1*), ∆Helo_4248 (*mscS2*), ∆Helo_3171 (*mscS3*) | This study |
|  | MSC-1324 | ∆Helo_2045 (*mscK*), ∆Helo_3378 (*mscS1*), ∆Helo_4248 (*mscS2*), ∆Helo_3171 (*mscS3*) | This study |
|  | MSC-RK | Helo_2045^+^, ∆Helo_3171, ∆Helo_3378, ∆Helo_4248 | This study |
|  | MSC‑R1 | Helo_3378^+^, ∆Helo_2045, ∆Helo_3171, ∆Helo_4248 | This study |
|  | MSC‑R2 | Helo_4248^+^, ∆Helo_2045, ∆Helo_3171, ∆Helo_3378 | This study |
|  | MSC‑R3 | Helo_3171^+^, ∆Helo_2045, ∆Helo_3378, ∆Helo_4248 | This study |
|  |  |  |  |

Suppl. Table 2

Plasmids used and generated in this work

| Plasmid | Usage | Reference |
| --- | --- | --- |
| pK18*mobSacB* | Homologous recombination in *H. elongata* | (5) |
| pK19*mobSacB* | Homologous recombination in *H.*elongata | (5) |
| pK18*mobSacB*::Helo_2045 | Deletion of Helo_2045 | This study |
| pK18*mobSacB*:: Helo_3171 | Deletion of Helo_3171 | This study |
| pK18*mobSacB*:: Helo_3378 | Deletion of Helo_3378 | This study |
| pK19*mobSacB*:: Helo_4248 | Deletion of Helo_4248 | This study |
| pK19*mobSacB*2045R | Re-Insertion of Helo_2045 | This study |
| pK19*mobSacB*3171R | Re-Insertion of Helo_3171 | This study |
| pK19*mobSacB*3378R | Re-Insertion of Helo_3378 | This study |
| pK19*mobSacB*4248R | Re-Insertion of Helo_4248 | This study |

Suppl. Table 3

PCR Primers used in this study.

All oligonucleotides utilized to construct plasmid vectors for the deletion/insertion of genes and for verification purposes are listed below.

| Name | Sequence | Application/Source |
| --- | --- | --- |
| M13 fwd | CGTTGTAAAACGACGGCCAGT | Screening of vectors |
| M13 rev | CACACAGGAAACAGCTATGACATG | Screening of vectors |
| 2045-1 fwd | GTACCCGGGGATCCTCTAGATCATAGTCGATCAGGCTGTCCC | Deletion/Re-insertion of Helo_2045 |
| 2045-1 rev | AGGGCTCCGCCGTCAAACGCGGTAGAAACGGCGTCCTATACCTCGGC | Deletion of Helo_2045 |
| 2045-2 fwd | TATAGGACGCCGTTTCTACCGCGTTTGACGGCGGAGCCC | Deletion of Helo_2045 |
| 2045-2 rev | CGACGGCCAGTGCCAAGCTCCAGCACAGACACATCCGTCC | Deletion/Re-insertion of Helo_2045 |
| 3171-1 fwd | GTACCCGGGGATCCTCTAGAAGGCCTTCCCCCTTCACTTCCAC | Deletion/Re-insertion of Helo_3171 |
| 3171-1 rev | ACTCTCTGGATGGAGATCCGTAGCGGCCCATTTCCGGTCTTTCC | Deletion of Helo_3171 |
| 3171-2 fwd | AGACCGGAAATGGGCCGCTACGGATCTCCATCCAGAGAGTCACG | Deletion of Helo_3171 |
| 3171-2 rev | CGACGGCCAGTGCCAAGCTTGGTGTCCGGCGC | Deletion/Re-insertion of Helo_3171 |
| 3378-1 fwd | GTACCCGGGGATCCTCTAGATGGTGGTGGGCATCGTCG | Deletion of Helo_3378 |
| 3378-1 rev | GCAGAAGCAAGGGAGGCGATGCAGCATTTCAGACTCTCGGTGC | Deletion of Helo_3378 |
| 3378-2 fwd | CCGAGAGTCTGAAATGCTGCATCGCCTCCCTTGCTTCTG | Deletion of Helo_3378 |
| 3378-2 rev | CGACGGCCAGTGCCAAGCTTGGCGAAGAGGTAGATGATGTGC | Deletion of Helo_3378 |
| 4248-1 fwd | GTACCCGGGGATCCTCTAGACACGACGGTGTGCCG | Deletion of Helo_4248 |
| 4248-1 rev | CGGCGAGGCCTGGCGGGAGGGCTATGGCCTCCGTGTCAGGGCG | Deletion of Helo_4248 |
| 4248-2 fwd | CCTGACACGGAGGCCATAGCCCTCCCGCCAGGCC | Deletion of Helo_4248 |
| 4248-2 rev | CGACGGCCAGTGCCAAGCTTCCAGCGTGGTCGATACCTATCT | Deletion of Helo_4248 |
| 3378Rfwd | AACAGCTATGACATGATTACAAGATGCTGCACAGCTACGG | Re-insertion of Helo_3378 |
| 3378Rrev | AGCTCGGTACCCGGGAATCATTACGTGCCCAACCTGAC | Re-insertion of Helo_3378 |
| 4248Rfwd | AACAGCTATGACATGATTACACCGTGGAGGCTTTATCGAG | Re-insertion of Helo_4248 |
| 4248Rrev | AGCTCGGTACCCGGGGCTGACCGAGGACAATCTG | Re-insertion of Helo_4248 |

Suppl. Table 4

Primers for qRT-PCR.

Oligonucleotides for qRT-PCR were designed with a length of 20 bp, Tm of 60 °C and GC-content ≤60 %. They produce fragments with a length of 100-120 bp.

| Target | Name | Sequence | Reference |
| --- | --- | --- | --- |
| Ref. gene | recA fwd | CGCCCTGAAGTTCTATTCCA | This study |
|  | rec A rev | GGCGCTACCTTGTTCTTGAC | This study |
| *msc* | 2045 fwd | CGGGAAATGAGCCTGGAGTT | This study |
|  | 2045 rev | CTGCAGTTGACGTACCTGGT | This study |
|  | 3171 fwd | GTCATGATCCCCAGCAGCTT | This study |
|  | 3171 rev | TGGCGATGTGAAGCTTTCCT | This study |
|  | 3378 fwd | AACATTCGTATCGGCTCGCT | This study |
|  | 3378 rev | TCTCCTCGGTCATCTCCCAG | This study |
|  | 4248 fwd | CCTGGTGATCGAACTGCTCA | This study |
|  | 4248 rev | GCGATCCAGCGATTCCAGTA | This study |

CLUSTAL format alignment by MAFFT FFT-NS-i (v7.429)

Helo_2045_core SVADAIVALLVVAMTLMLARNLPGLL-EVMVLSRLELKQGSAYAISSLLSYTIVGTGVVM

MSCK_ECOLI_core TMGSLLFAIIASMVAWALIRNLPGLL-EVLVLSRLNMRQGASYAITTILNYIIIAVGAMT

MSCM_ECOLI_core TLGAVLIAILVFIITTQLVRNLPALL-ELAILQHLDLTPGTGYAITTITKYLLMLIGGLV

Helo_3378_core SFAINLVAAIAIFVIGRWVAKLLHRL-VTKAMKRAKTDPLIVTFAGNIFYVLLMFAVVLA

MSCS_ECOLI_core SYAVNIVAALAIIIVGLIIARMISNA-VNRLMISRKIDATVADFLSALVRYGIIAFTLIA

YNAI_ECOLI_core FICTSLIAVILTIKLFLLINQF------EKQQIKKGRDITSARIMSRIIKITIIVVLVLL

YBDG_ECOLI_core QLWIMMYALLSVFSLLDVILNLAQKFPAASQLPLKGIFQGIKLIGAILVGILMISLL---

Helo_3171_core GFTILLAWLAWIFADTAIQRALVSSARSRGRRVNQARAQTITPMIRNVIFATIVIIAAIV

Helo_4248_core AVILLVALAIWLVLASLIEHKLNP---ATGSGEPSSRAQTLLSLFRNAIAITLITVTIMV

YBIO_ECOLI_core ALILFFSAVGWTVLASLIENRLASD--IHGRPLPSARTRTLLTLFRNALAVIISTITIMI

A0A072ZAU2_core RFLVKGQAKKTDIPPEVQIMR------KTQEQRRQARIRTLGAVGKSAVAIFVWTWAALA

Cgl1270_CORGL_c --------------MRIIKRRVESAA-DADTTKNQLAFAGVGVYIAQIVAFFMLAVSAM-

:

Helo_2045_core ALGTLGVSWSKLQWLVAALGVGLGFGLQEIFANFISGLIILFERPVRIGDTITL---G-N

MSCK_ECOLI_core VFGSLGVSWDKLQWLAAALSVGLGFGLQEIFGNFVSGLIILFERPVRIGDTVTI---G-S

MSCM_ECOLI_core GFSMIGIEWSKLQWLVAALGVGLGFGLQEIFANFISGLIILFEKPIRIGDTVTI---R-D

Helo_3378_core AIGQLGIQTTSLIAVLGAAGLAIGLALQGSLANFAAGVMVVLFRPYRVGDYIEG---G-G

MSCS_ECOLI_core ALGRVGVQTASVIAVLGAAGLAVGLALQGSLSNLAAGVLLVMFRPFRAGEYVDL---G-G

YNAI_ECOLI_core YGEHFGMSLSGLLTFGGIGGLAVGMAGKDILSNFFSGIMLYFDRPFSIGDWIRSPD-R-N

YBDG_ECOLI_core ----IGQSPAILISGLGAMAAVLMLVFKDPILGLVAGIQLSANDMLKLGDWLEMP--KYG

Helo_3171_core GLANLGVNVTPLLAGAGVIGLAIGFGAQTLVQDLITGIFILIEDSLAVDDFVKI---N-G

Helo_4248_core VLAEIGINIGPLIAGAGVLGLAIGFGAQKLVQDIITGVFIQVENALNVGDVVTL---G-G

YBIO_ECOLI_core VLSEIGVNIAPLLAGAGALGLAISFGSQTLVKDIITGVFIQFENGMNTGDLVTI---G-P

A0A072ZAU2_core ILTEIGLNVAPLIASAGVAGVALGFGAQSLVKDFLSGIFMLIEDQYGVGDTIDV---GDG

Cgl1270_CORGL_c --QAFGFSLAGAAIPATIASAAIGLGAQSIVADFLAGFFILTEKQFGVGDWVRFEGNGIV

.* . . : : : . .: :*. : .: :

Helo_2045_core LTGTVSRIRIRATTVTDFDRKEIIIPNKTFVTDQLINWS--LSDNITRVILTYGV-----

MSCK_ECOLI_core FSGTVSKIRIRATTITDFDRKEVIIPNKAFVTERLINWS--LTDTTTRLVIRLGV-----

MSCM_ECOLI_core LTGSVTKINTRATTISDWDRKEIIVPNKAFITEQFINWS--LSDSVTRVVLTIPA-----

Helo_3378_core VSGTVEEVQIFTTELSTPDNRKIIVPNGQMLSDAITNYS---AHATRRVDLVVGV-----

MSCS_ECOLI_core VAGTVLSVQIFSTTMRTADGKIIVIPNGKIIAGNIINFS---REPVRRNEFIIGV-----

YNAI_ECOLI_core IEGTVAEIGWRITKITTFDNRPLYVPNSLFSSISVENPG---RMTNRRITTTIGL-----

YBDG_ECOLI_core ADGAVIDIGLTTVKVRNWDNTITTIPTWSLVSDSFKNWSGMSASGGRRIKRSISIDVTSI

Helo_3171_core HMGTVEGLTLRTVRLRDLDGIVHIITFSRI--DSIHNMS--RQFGIALMKIRIPY-----

Helo_4248_core ITGTAERLSIRSVGLRDLSGTYHIVPFSSV--DTVSNYM--REFAYHVGEYGIAY-----

YBIO_ECOLI_core LTGTVERMSIRSVGVRQDTGAYHIIPWSSI--TTFANFV--RGIGSVVANYDVDR-----

A0A072ZAU2_core IIGDVEDISLRTTTLRDLDGTVWYIRNGEI--LRVGNFS--NEYAIARFEVPVGL-----

Cgl1270_CORGL_c VEGTVIEITMRATKIRTIAQETVIIPNSTA--KVCINNS--NNWSRAVVVIPIPML----

* . : . : : *

Helo_2045_core --------------------------------------------ELGSDHRLVQRLLQQA

MSCK_ECOLI_core --------------------------------------------AYGSDLEKVRKVLLKA

MSCM_ECOLI_core --------------------------------------------PADANSEEVTEILLTA

Helo_3378_core --------------------------------------------GYGDDIDTVRRVLEGV

MSCS_ECOLI_core --------------------------------------------AYDSDIDQVKQILTNI

YNAI_ECOLI_core ------------------------------------------RYEDAAKVGVIVEAVREM

YBDG_ECOLI_core RFLDEDEMQRLNKAHLLKPYLTSRHQEINEWNRQQGSTESVLNLRRMTNIGTFRAYLNEY

Helo_3171_core ------------------------------------------DMKIDDAITLMHETARAL

Helo_4248_core ------------------------------------------RENIDEAIQALHAAFDDL

YBIO_ECOLI_core ------------------------------------------HEDADKANQALKDAVAEL

A0A072ZAU2_core ------------------------------------------SNDSDRAWDVIENSFQEA

Cgl1270_CORGL_c -----------------------------------------GSENITDVIARSEAATRRA

Helo_2045_core ADENG-------RVLRDPAPETFFMRYSANAMEFELRIFVNSL--GDRLYATDELNGRVG

MSCK_ECOLI_core ATEHP-------RVMHEPMPEVFFTAFGASTLDHELRLYVREL--RDRSRTVDELNRTID

MSCM_ECOLI_core ARRCS-------LVIDNPAPEVFLVDLQQGIQIFELRIYAAEM--GHRMPLRHEIHQLIL

Helo_3378_core VADDA-------RVLADPAPNIRIGSLGDSSVNWIVRPWVKA---ADYWDVYWEMTEEIK

MSCS_ECOLI_core IQSED-------RILKDREMTVRLNELGASSINFVVRVWSNS---GDLQNVYWDVLERIK

YNAI_ECOLI_core LKNHP-------AIDQRQTLLVYFNQFADSSLNIMVYCFTKTTVWAEWLAAQQDVYLKII

YBDG_ECOLI_core LRNHP-------RIRKDMTLMVRQLAPGDNGLPLEIYAFTNTVVWLEYESIQADIFDHIF

Helo_3171_core RQDPMMR-----HHIWSPLEMQGIHAFEDGCPILRMRFRTAP---EMQWDVSRAFNLLLK

Helo_4248_core SEDEEQK-----ANLLEPLEVAGVIALADSSVNIRVRIKTTP---GTQWAVGRAYNRLVK

YBIO_ECOLI_core MENEEIR-----GLIIGEPNFAGIVGLSNTAFTLRVSFTTLP---LKQWTVRFALDSQVK

A0A072ZAU2_core VKMEAIK-----DSVIDTPEMKGISAFEPDHMTFRGVVKTLP---GYQWEVQRYVYAKVL

Cgl1270_CORGL_c LGQEKIAPEILGELDVHPATEVTPPTVVGMPWMVTMRFLVQVT-AGNQWLVERAIRTEII

:

Helo_2045_core ELLAEHDLDIAFNQMDVWLH

MSCK_ECOLI_core QLCRENDINIAFNQLEVHLH

MSCM_ECOLI_core AGFHAHGIDMPFPPFQMRLE

Helo_3378_core RRFDREGINIPFPQRDVHVY

MSCS_ECOLI_core REFDAAGISFPYPQMDVNFK

YNAI_ECOLI_core DIVQSHGADFAFPSQTLYMD

YBDG_ECOLI_core AIVEEFGLRLHQSPTGNDIR

Helo_3171_core QRMEEQEIDLGVPRLSVSME

Helo_4248_core YHFDDAGIEIPFPHTTLYFG

YBIO_ECOLI_core KHFDLAGVRAPVQTYQVLPA

A0A072ZAU2_core SDMQKEGITTPYPHGMGGVG

Cgl1270_CORGL_c SEFWEEYGSATTTSGTLIDS

Suppl. Figure 1

Alignment of the mscS core region of the analyzed proteins. Included in this analysis are the 4 MscS proteins from *H. elongata*, 6 mscS-related proteins from *E. coli*, and two MscS-related proteins from *C. glutamicum*. The MscS core region was selected for this analysis (for the positions of the core region of the *H. elongata* proteins see Table 1). It should be noted that YbdG has an insert of ca 50 amino acids, compared to the other MscS-related proteins. This may correlate with the absence of channel activity of this protein (PMID:22874652, PMID:31256002)

CLUSTAL format alignment by MAFFT FFT-NS-i (v7.429)

YBIO_ECOLI_N M----------------RWILFILFCLLGAPAHAVSIPGVTTTTTTDSTTEPAPEPDIEQ

Helo_4248_N MSKTRLLP------LALGWLVIALLAFAGPALAQSSDPGAS-----------AP------

Helo_3171_N M-----LPIAHRYSLALRGLLMLLLLALPLLVAAQPSPLSGLANAKQSQGH-DP----QQ

* ::: *: . * *

YBIO_ECOLI_N KKAAYGALADVLDNDTSRKELIDQLRTVA-ATPPAEPVPKIVPPT-----LVEEQTVLQK

Helo_4248_N ---ANAALADMLENPETRQQLIDQLRGMADAAESSGEQASTAPP-------VKDPSLPRQ

Helo_3171_N LQASLDQVIATLQDDAKRQELLESLEALRQANQPAAEEESFTSPQGLLGALADTFNQLGE

: : *:: .*::*::.*. : * .: . ..* .. . :

YBIO_ECOLI_N VTEVSRHYGEALSARFGQLYRNIT------GSPHKPFNPQTFSNALTHFSMLAVLVFGFY

Helo_4248_N LAEFTSHVAGDIGLQVGLLYGTLTHLFT-GGDEGASIDLAVVTSAAINLGLVILATFALF

Helo_3171_N QAESGRSPLDGWRLQLTEGWQDLTELVVRTGN-------AEIVRFIIELTVLVAIWGGLL

:* :. : :* *. . .: :: .:

YBIO_ECOLI_N WLIRLCALPLYRKMGQWARQKNRERSNWLQLPAMIIGAFIIDLLLLALTLFVGQVLS---

Helo_4248_N LIIRRLVRPVFTRLSLWSMNGPALQP-LLRLVICVVLAALVDVLVVALAYIGGNLLA---

Helo_3171_N VIFIALGRMLARRRG-WPLDLPREPR-AHLLAIHFLRRMLPWALAFALTMGIAQLVSGSA

:: : : . *. : * .: : * .**: .::::

YBIO_ECOLI_N --------DNLNAGSRTIAFQQSLFLNAFALIEFFKAVLRLIFCPNVAELRP-FTIQDES

Helo_4248_N --------TFVVGESGALTTRASLFLNAFLVIELLKAGVRMLFASRYEGLRL-LPVNAED

Helo_3171_N GRTGALVIAYVALCGRTLSVVVETFISVFTRGHRFVA-VRIL---QNHGLRPLFMIGALI

: . ::: . *:..* . : * :*:: . ** : :

YBIO_ECOLI_N ARYWSRRLSWLSSLIGYGLIVAVPIISNQVNVQIGALANVIIMLCMTVWALYLIFRNKKE

Helo_4248_N ASYWNRWIARLIGLAGYGLMVVVPLISAYLSPTLGQSVGTLIMIGAFIYAVAVVLRNRQR

Helo_3171_N ALGDALNSSRLTDLIGLDLASLCSVLANTLAAVLSGR-----FILNFKRPIKHLIRNR--

* : * .* * .* .::: : :. :: .: ::**:

YBIO_ECOLI_N ITQHL-LNFAEHSLAFFSLFIRAFALVWH---WLASAYFIVLFFFSLFDPGNSLKFMMGA

Helo_4248_N LRDSLHRQAARASLSASRVALTLLGRTWH---LLAIAYFFMVLVLTLTRPADALPFVLLA

Helo_3171_N ------PYSHRRDKGSASEVIRVVGGLWHVPALLLVGGSLLAIFVTGGDVGTAL----AR

. . . : .. ** * . :: :..: . :*

YBIO_ECOLI_N TVRSLAIIGIAAFVSGMFSRWLAKTITLSPHTQRNYPELQKRLNGWLSAALKTARILTVC

Helo_4248_N TLETLAAVLVGLGASKFLTQTIGRRITLQDDLRQKLPLLEARLNAYIPKALRVVRIVLLV

Helo_3171_N SIISAALLVLTLVVAGLLRRHAEKR-----SKRRRQSEYRRRLERF---GYALGHVLAWF

:: : * : : .: :: : : ::. . . **: : . :::

YBIO_ECOLI_N VAVMLLLSAWGLFDFWNWLQNGAGQKTVDILIRI

Helo_4248_N LVVMVSLNAWQVFDLAGWYASEAGRGLVARLFSI

Helo_3171_N AFAELSLQVWGASLIGIGTQGVASVRIGQALLGI

. : *..* : . *. *: *

Suppl. Figure 2

Alignment of the N-terminal regions of *E. coli* YbiO and its two *H. elongata* homologs. No InterPro domains have been assigned to this region of approximately 450 amino acids (see Table 1).

CLUSTAL format alignment by MAFFT FFT-NS-i (v7.429)

Helo_2045_N MPVRQVNCGGVATPAIALSAICIENHKNFRGVTDDEVFVNVVSRRVGMKVLHIVCLLVAL

MSCK_ECOLI_N MTMFQY----------------YKRSRHF-------VFSAF-----------IAFVFVLL

*.: * :. ::* ** . *. ::* *

Helo_2045_N LGSTAWAV-----EPPTREAIEQRLEELRPADGKEPDAAAQEKIDTLQAALEDLAAKEAA

MSCK_ECOLI_N CQNTAFARASSNGDLPTKADLQAQLDSL----NKQKDLSAQDKL--VQQDLTDTLATLDK

.**:* : **: :: :*:.* .*: * :**:*: :* * * *.

Helo_2045_N KARLED----LESRVEKAPAELRELQQALSENQD-DTPAASLEALESLDLETLEIRLKEA

MSCK_ECOLI_N IDRIKEETVQLRQKVAEAPEKMRQATAALTALSDVDNDEETRKILSTLSLRQLETRVAQA

*::: *..:* :** ::*: **: .* *. : : *.:*.*. ** *: :*

Helo_2045_N SAALRRDQDRLSQIETRLLGTQTLPERAQQGISDATQAVEESRRTLEDLAARDVDESDPR

MSCK_ECOLI_N LDDLQNAQNDLASYNSQLVSLQTQPERVQNAMYNASQQLQQIRSRLD---GTDVGETALR

*:. *: *:. :::*:. ** ***.*:.: :*:* ::: * *: . **.*: *

Helo_2045_N ---HMRARTQRALAEQRLALYQRELATNSRLRELAQQRRDLLERRVTTQEAKVLTLQRLV

MSCK_ECOLI_N PSQKVLMQAQQALLNAEIDQQRKSLEGNTVLQDTLQKQRDYVTANSARLEHQLQLLQEAV

:: ::*:** : .: ::.* *: *:: *::** : . : * :: **. *

Helo_2045_N DQRRRERSEQAIAEAVQ-DEPDDVASHPLVNEAQKANREMSLELLRVTSRANELVRQGLE

MSCK_ECOLI_N NSKRLTLTEKTAQEAVSPDEAARIQANPLVKQELEINQQLSQRLITATENGNQLMQQNIK

:.:* :*:: ***. **. : ::***:: : *:::* .*: .*...*:*::*.::

Helo_2045_N VRRQLDQVRQLQRGMDEHVEAIRGSTLLSRILRELRQALPKVEVRGGLKDEIADWRLRQF

MSCK_ECOLI_N VKNWLERALQSERNIKEQIAVLKGSLLLSRILYQQQQTLPSADELENMTNRIADLRLEQF

*:. *::. * :*.:.*:: .::** ****** : :*:**..: .:.:.*** **.**

Helo_2045_N ELDRQRETLKDAEALARKRMESAAGEEVSSALVDPLAQLFRARRDLLDQLEPTYGEMLST

MSCK_ECOLI_N EVNQQRDALFQSDAFVNK-LEEGHTNEVNSEVHDALLQVVDMRRELLDQLNKQLGNQLMM

*:::**::* :::*:..* :*.. :**.* : *.* *:. **:*****: *: *

Helo_2045_N AIELQLHQQQLLTTSRSLRDTIDKQLFWVANARPLDLAWLLKLPDHLRTEWREGE----W

MSCK_ECOLI_N AINLQINQQQLMSVSKNLKSILTQQIFWVNSNRPMDWDWIKAFPQSLKDEFKSMKITVNW

**:**::****::.*:.*:. : :*:*** . **:* *: :*: *: *::. : *

Helo_2045_N RHALPNHWTMPDSGALLGVPLLLAAAGLLLLRRT-LERRLLKLHDEIGHLRRDSQAHTPK

MSCK_ECOLI_N QKAWPAVFI----AFLAGLPLLL-IAGLIHWRLGWLKAYQQKLASAVGSLRNDSQLNTPK

::* * : . * *:**** ***: * *: ** . :* **.*** :***

Helo_2045_N AILFNALLAMPMPLILAGIGLALLFGGQGVARDIGWSLA-QIALAWAVVAWARRLLTADG

MSCK_ECOLI_N AILIDLIRALPVCLIILAVGLILLTMQLNIS-ELLWSFSKKLAIFWLVFGLCWKVLEKNG

***:: : *:*: **: .:** ** .:: :: **:: ::*: * *.. . ::* :*

Helo_2045_N VAVRQFYWPPAYVAVLKRWLFWLLASMIPVLLVAPPARDAGINLNYRPLAMTVLLAGFLG

MSCK_ECOLI_N VAVRHFGMPEQQTSHWRRQIVRISLALLPIHFWSVVAELSPLHLMDDVLGQAMIFFNLLL

****:* * .: :* :. : :::*: : : *. : ::* *. :::: .:*

Helo_2045_N MSLALTKLIM-------AHTPFFGVKFFRLVLGLAMAAVPLLLGGLVVSGYAYTALSLVS

MSCK_ECOLI_N IAFLVWPMCRESWRDKESHT-------MRLVTITVLSIIPIALMVLTATGYFYTTLRLAG

::: : : :** :*** .:: :*: * *..:** **:* *..

Helo_2045_N RFMITLYVLGVWILVEAAVVRGLAVAARRLAYRRALARRRALQEHEGESGLDVVEEPPLD

MSCK_ECOLI_N RWIETVYLVIIWNLLYQTVLRGLSVAARRIAWRRALARRQNLVK-EGAEGAEPPEEPTIA

*:: *:*:: :* *: :*:***:*****:*:*******: * : ** .* : ***.:

Helo_2045_N MQQVNQQSLRLAKLALLIGFLVVLYLVWADLLTVLGYLEQVMLLGGDGEQGADLVGGAV

MSCK_ECOLI_N LEQVNQQTLRITMLLMFALFGVMFWAIWSDLITVFSYLDSITLWHYNGTEAGAAVVKNV

::*****:**:: * :: * *::: :*:**:**:.**:.: * :* :.. * *

Suppl. Figure 3

Alignment of the N-terminal regions of *E. coli* MscK and its *H. elongata* homolog. This region contains two assigned InterPro domains: IPR024393 “MscS_porin” and IPR025692 “MscS_IM_dom1” (see Table 1).

|  |  | Down-shock | Up-shock |  |
| --- | --- | --- | --- | --- |
| *mscK* | normalized relative gene expression |  |  |  |
| *mscS1* |  |  |  |  |
| *mscS2* |  |  |  |  |
| *mscS3* |  |   t [min] |   t [min] |  |
| Suppl. Figure 4  Relative gene expression of all MS channels before and after an osmotic shock. *H. elongata* wildtype was exposed to osmotic down-shock (1 M NaCl to 0.1 M NaCl) and osmotic up-shock (1 M NaCl to 2 M NaCl). Cultures were grown in MM63 medium. Gene expression was assessed 30 min and 15 min before and after the shock. Astérisques next to the graph marks significant difference between expression after down- and upshock with p-values <0.05. Data presented are from three independent experiments (three biological replicates). Each experiment was measured three times (three technical replicates). | | | | |

REFRENCES FOR SUPPLEMENTAL MATERIAL

1. Hanahan D. 1983. Studies on transformation of *Escherichia coli* with plasmids. J Mol Biol 166:557-80.

2. Thoma S, Schobert M. 2009. An improved *Escherichia coli* donor strain for diparental mating. FEMS Microbiol Lett 294:127-32.

3. Vreeland RH, Litchfield CD, Martin EL, Elliot E. 1980. *Halomonas elongata*, a new genus and species of extremely salt-tolerant bacteria. Int J Syst Bacteriol 30:485-495.

4. Schwibbert K, Marin-Sanguino A, Bagyan I, Heidrich G, Lentzen G, Seitz H, Rampp M, Schuster SC, Klenk HP, Pfeiffer F, Oesterhelt D, Kunte HJ. 2011. A blueprint of ectoine metabolism from the genome of the industrial producer *Halomonas elongata* DSM 2581^T^. Environ Microbiol 13:1973-1994.

5. Schäfer A, Tauch A, Jäger W, Kalinowski J, Thierbach G, Pühler A. 1994. Small mobilizable multi-purpose cloning vectors derived from the *Escherichia coli* plasmids pK18 and pK19: selection of defined deletions in the chromosome of *Corynebacterium glutamicum*. Gene 145:69-73.
